# Anticholinesterase Inhibitory Activity of Quaternary Alkaloids from *Tinospora crispa*

**DOI:** 10.3390/molecules19011201

**Published:** 2014-01-20

**Authors:** Mashitah Yusoff, Hazrulrizawati Hamid, Peter Houghton

**Affiliations:** 1 Faculty of Industrial Sciences & Technology, Universiti Malaysia Pahang, Lebuhraya Tun Razak, Gambang Kuantan, Pahang 26300, Malaysia; E-Mail: hazrulrizawati@ump.edu.my; 2 Department of Pharmacy, King’s College London, Franklin-Wilkins Building, 150 Stamford Street, London SE1 8WA, UK; E-Mail: peter.houghton@kcl.ac.uk

**Keywords:** *Tinospora crispa*, Menispermaceae, ethnoremedy, anticholinesterase inhibitory activities, quaternary alkaloids, 4,13-dihydroxy-2,8,9-trimethoxydibenzo[a,g]quinolizinium, dihydrodiscretamine, columbamine

## Abstract

Quaternary alkaloids are the major alkaloids isolated from *Tinospora* species. A previous study pointed to the necessary presence of quaternary nitrogens for strong acetylcholinesterase (AChE) inhibitory activity in such alkaloids. Repeated column chromatography of the vine of *Tinospora crispa* extract led to the isolation of one new protoberberine alkaloid, 4,13-dihydroxy-2,8,9-trimethoxydibenzo[a,g]quinolizinium (**1**), along with six known alkaloids—dihydrodiscretamine (**2**), columbamine (**3**), magnoflorine (**4**), *N*-formylannonaine (**5**), *N*-formylnornuciferine (**6**), and *N*-*trans*-feruloyltyramine (**7**). The seven compounds were isolated and structurally elucidated by spectroscopic analysis. Two known alkaloids, namely, dihydrodiscretamine and columbamine are reported for the first time for this plant. The compounds were tested for AChE inhibitory activity using Ellman’s method. In the AChE inhibition assay, only columbamine (**3**) showed strong activity with IC_50_ 48.1 µM. The structure–activity relationships derived from these results suggest that the quaternary nitrogen in the skeleton has some effect, but that a high degree of methoxylation is more important for acetylcholinesterase inhibition.

## 1. Introduction

Quaternary alkaloids are the major alkaloids isolated from *Tinospora* species [1,2,3]. A previous study attributed the strong acetylcholinesterase (AChE) inhibitory activity observed in tertiary alkaloids to the ability of the nitrogen present to become protonated and bind to the oxyanion hole in the active site of the enzyme [4]. Berberine was recently considered as an attractive compound for Alzheimer disease treatment so berberine-based derivatives were developed to prepare more potent AChE inhibitors [5]. The related compounds pseudoberberine and pseudocoptisine have been shown to alleviate scopolamine-induced memory impairment in *in vivo* models [5,6].

*Tinospora crispa* (L.) Hook.f. & Thomson (family: Menispermaceae) is widely used as Malay and Thai ethnoremedies for the treatment of hypertension and diabetes [7]. Previous phytochemical investigations have shown that quaternary alkaloids are the major alkaloids present in *Tinospora* species, and they are mostly of the protoberberine type. *N*-acylaporphine alkaloids are non-quaternary alkaloids which have also been isolated from certain *Tinospora* species [8].

**Figure 1:**
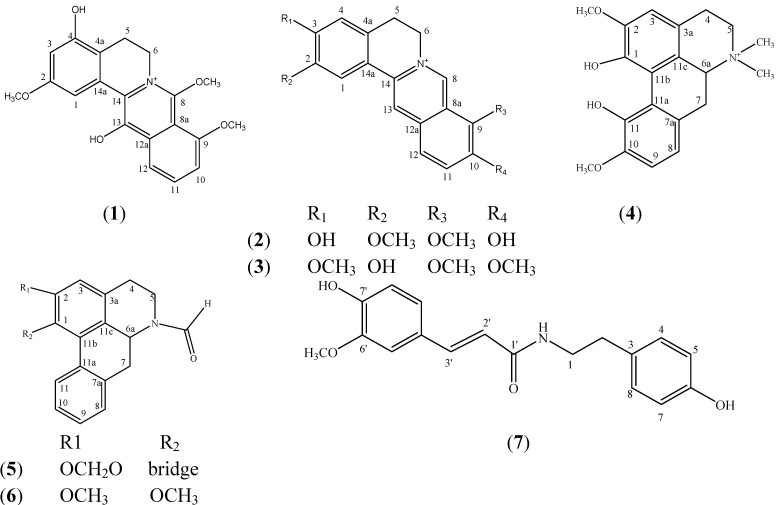
(**1**) 4,13-Dihydroxy-2,8,9-trimethoxydibenzo[a,g]quinolizinium; (**2**) dihydro-discretamine; (**3**) columbamine; (**4**) magnoflorine; (**5**) *N*–formylannonaine; (**6**) *N*-formylnornuciferine; and (**7**) *N*-*trans*-feruloyltyramine.

In this paper, we report the isolation and structural elucidation of a new, fully aromatic protoberberine alkaloid, 4,13-dihydroxy-2,8,9-trimethoxydibenzo[a,g]quinolizinium (**1**) along with six known alkaloids—dihydrodiscretamine (**2**) columbamine (**3**) magnoflorine (**4**) *N*-formylannonaine (**5**), *N*-formylnornuciferine (**6**) and *N*-*trans*-feruloyltyramine (**7**) (Figure 1). Compound **1** is a novel protoberberine alkaloid, whereas compounds **2** and **3** were found for the first time in this plant. The AChE inhibition activities of all seven compounds are also reported.

## 2. Results and Discussion

Compound **1** was a brown gum and was shown to have the molecular formula [C_20_H_18_NO_5_]^+^ based on the HREIMS. In the mass spectrum of **1**, the *m/z* peak 310.0366 has virtual maximum intensity (100%), which indicates a high stability of the ion [C_17_H_17_NO_5_]^+^ with respect to electron impact. Similar fragmentation processes involving direct elimination of CH_2_ from the molecular ion with *m/z* 352.0075 to *m/z* 310.0366 characterized the presence of three methoxy groups in the sample. The ion at *m/z* 337.0475 was determined as the neutral loss of the radical group radical CH_3_∙ from C-9. The ion at *m/z* 308.0654 can be produced by loss of the carbonyl group formed in C-9 of the D ring via molecular rearrangement. The fragmentation patterns of these compounds are similar to those of protoberberine compounds [9]. The UV spectrum showed maximal absorptions at 213 and 291 nm, suggesting the presence of a highly conjugated aromatic system. The IR spectrum showed absorptions at 3449 cm^−1^ (broad) due to hydroxyl group and at 1091 cm^−1^ due to C-O. Analysis of the ^1^H-NMR, ^13^C-NMR, DEPTQ, and HMQC data (Table 1) of **1** revealed the presence of a fully aromatic skeleton of protoberberine with seven aromatic protons. Two *ortho*-coupled aromatic protons at δ 6.52 and δ 6.86 (*J* = 8.0, 2.0 and *J* = 8.0, respectively) could only be bonded to C-11 and C-12. Another aromatic proton at δ 6.56, with *meta* coupling constant *J* = 2.0, could be bonded to C-10. The ^1^H spectrum revealed pyridine protons at δ 7.96 and 7.87 (*J* = 6.0 and *J* = 6.5, respectively) assigned to H-5 and H-6 of the skeleton. Two broad singlet aromatic protons at δ 7.35 and 7.35 were assigned to H-4 and H-1. The COSY NMR spectrum revealed the correlations H5 to H6, H10 to H11, and H11 to H12. The ^13^C NMR and DEPTQ spectra revealed the presence of twenty carbon atoms, including methine at δ 107.69 (C-1), 103.89 (C-3), 121.33 (C-5), 130.32 (C-6), 114.86 (C-10), 118.52 (C-11), and 111.85 (C-12); methoxy at δ 55.08 (2-OCH_3_), 54.94 (9-OCH_3_), and 44.17 (8-OCH_3_); and ten quaternary carbons at 127.21 (C-4a), 127.19 (C-12a), 132.05 (C-14a), 136.98 (C-14), 147.22 (C-9), 147.55 (C-13), 150.46 (C-8), 162.37 (C-2), and 163.81 (C-4). In **1**, an electron donor substituent, the 13-hydroxyl group, is *para* to the 8-methoxy group, thus inhibiting the electronic resonance. A shielding effect is observed for the 8-methoxy ^13^C chemical shift [10]. If a substituent were *para* to the methoxy group, then it could interact with the methoxy group only by electronic resonance through the π-system. As the hydroxyl groups have opposite electronic characteristics, they will affect the resonance interaction of the methoxy ^13^C chemical shifts [10]. According to [11], when two atoms of the same molecule are in close proximity to one another, a shielding effect is observed on both if the van der Waals forces yield an attractive interaction, whereas a deshielding effect is observed if there is a repulsive interaction. These considerations suggest that the observed shielding effect is due to an attractive interaction.

**Figure 2:**
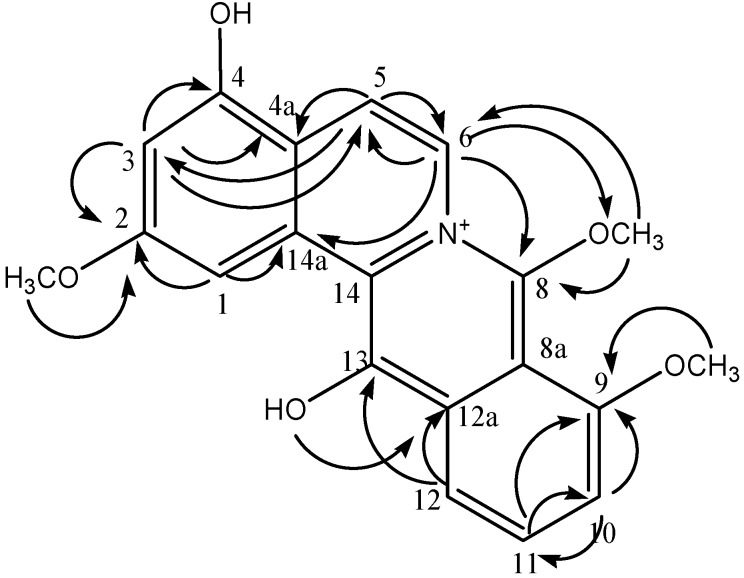
HMBC correlation of (**1**).

To confirm the distribution of methoxy and hydroxy groups, HMBC experiments were executed (Figure 2). The assignment of the 8-methoxy group at δ 4.20 was confirmed by its correlation with signals at 150.46 (C-8) and 130.32 (C-6). The position of the 9-methoxy group at δ 3.81 was assigned based on the correlation of the proton at δ 6.56 (H-10) with 147.22 (C-9) and 118.52 (C-11). 

The position of the 2-methoxy group was assigned based on the correlation of H-1 to neighboring carbons at 162.37 (C-2), 132.05 (C-14a), and 150.46 (C-8). The position of the hydroxyl at δ 4.71 was assigned based on its correlation to 127.19 (C-12a). The ^1^H-NMR and ^13^C-NMR data of **1** are closely related to those of synthetic dibenzo[a,g]quinolizinium compounds [12].

**Table 1:** ^1^H (500 MHz, MeOD), ^13^C (125 MHz, MeOD), DEPTQ, COSY, HMQC and HMBC of **1**.

Carbons	δC (ppm)	δH (ppm) Int. Mult. J	^1^H-^1^H COSY	HMBC
1	107.69	7.28 (1H, br s)	-	C14a, C2
2	162.37	-	-	-
3	103.89	7.35 (1H, br s)	-	C5, C4a, C4, C2
4	163.81	-	-	-
4a	127.21	-	-	-
5	121.33	7.87 (1H, d, 6.50)	H6	C3, C4a, C6
6	130.32	7.96 (1H, d, 6.00)	H5	C8, C14a, C5, 8-OCH_3_
8	150.46		-	-
8a	121.53	-	-	-
9	147.22		-	-
10	114.86	6.56 (1H, d, 2.0)	H11	C11, C9
11	118.52	6.52 (1H, dd, 8.0, 2.0)	H12	C9, C10
12	111.85	6.86 (1H, d, 8.5)	-	C12a, C13
12a	127.19	-	-	-
13	147.55	-	-	-
14	136.98	-	-	-
14a	132.05	-	-	-
2-OCH_3_	55.08	4.07 (3H, s)	-	C2
9-OCH_3_	54.94	3.81 (3H, s)	-	C9
8-OCH_3_	44.17 ^a^	4.20 (3H, s)	-	C8, C6
13-OH	-	4.71 (1H, s)	-	C12a

^a^ may be interchangeable.

The two known compounds were identified as dihydrodiscretamine (**2**) and columbamine (**3**) by comparing their physical data (m.p., MS, ^1^H-, and ^13^C-NMR) with those reported in the literature [3,13]. Magnoflorine (**4**) [14], *N*-formylannonaine (**5**) [15], *N*-formylnornuciferine (**6**) [15] and *N*-*trans*-feruloyltyramine (**7**) [16,17] were similarly identified.

The *in vitro* AChE inhibitory activities of compounds **1**–**7** (Table 2) are represented as concentration (μM) that inhibits 50% of the AChE activity (IC_50_). The IC_50_ values are the mean ± standard deviations of two independent experiments. Physostigmine was used as a positive control. In order to classify the AChE inhibitors, the minimum concentration for the strongest activity is IC_50_ < 150 μM and for moderate activity IC_50_ 200–500 μM. Concentrations of AChE inhibitory activity >500 µM are considered inactive [18]. Compound **3** was found to exhibit the strongest AChE inhibition activity with IC_50_ value close to that of physostigmine, the positive control. Compounds **2** and **5** showed moderate AChE inhibitory activity in microplate assays. The efficacy of inhibition of AChE was found to decrease in the order of **1** > **6**. Compounds **1**, **4**, **6** and **7** showed very weak or no inhibitory activity.

**Table 2:** Acetylcholinesterase inhibitory activity (IC_50_ values) of the isolated alkaloids.

Compound	IC_50_ (μM)
1	517.6 ± 5.3
2	276.1 ± 1.8
3	48.1 ± 1.3
4	NA
5	415.3 ± 2.7
6	564.6 ± 2.1
7	NA
Physostigmine	31.4 ± 0.5

Many natural tertiary alkaloids have been studied as AChE inhibitors [19], but relatively few quaternary alkaloids. Tertiary alkaloids are considered to become protonated at physiological pH to afford quaternary centres, and thus mimic the quaternary nitrogen of the well-known quaternary nitrogen inhibitors of cholinesterases such as decamethonium [20], which binds to the oxyanion part of the active site of acetylcholinesterase.

The quaternary ammonium head groups in protonated and quaternary alkaloids are able to bind via cation–π and lipohilic interactions to the tryptophan residues and the aromatic gorge [21]. It is generally believed that, due to the presence of the rings of the aromatic amino acids in AChE gorges, the cationic ligands move toward the active sites by diffusion on the surface of the enzyme [20]. The active compound **3** is a quaternary alkaloid and therefore a positive charge of these compounds may facilitate the diffusion to the aromatic gorge. This finding is in agreement with a proposal by [22] that reveal a series of novel berberine derivatives as potent AChE inhibitors.

However, the structure–activity relationships deduced from our results indicate that a permanent positive charge on the nitrogen in quaternary alkaloids is not a guarantee of inhibitory activity, since some of the compounds were found to be inactive. A lipophilicity linked with a high degree of methoxylation, as seen in **3** plays a more important part in determining high activity.

Conversely, compound **1** is a quaternary alkaloid, but it exhibits poor inhibition.The presence of two hydroxyl-functionalities provides a donor and an acceptor potential for hydrogen bonding and thus the most polar derivatives tested showed the weakest inhibitory potential. The introduced free electron pairs at the oxygen atom of the hydroxyl group are only weak donors or acceptors for π-π interactions. It is more likely that they are involved in strong hydrogen bonding interactions to water molecules, making compounds even more hydrophilic [21]. The lack of aromatic π–π interaction potential provides the lowest lipophilic interaction potential and accounts for the observed low inhibitory effect.

Compounds with aromatic rings showed better activity than those without aromatic rings, e.g., **5** and **6**. The moderately and lower AChE inhibitory activity of the aporphine alkaloids could be due to the lack of ring double bonds. Derivatives lacking ring double bonds demonstrated a decrease in activity, indicating the significance of an aromatic planar moiety in interactions with enzyme-binding sites. Flexibility, however, may be disadvantageous for binding affinity [17].

## 3. Experimental

### 3.1. General

Ultraviolet (UV) spectra were recorded on a Genesys 10S UV-VIS (Thermo Fisher Scientific, Madison, WI, USA) visible spectrophotometer in methanol solution. Infrared (IR) spectra were recorded on a Perkin Elmer 100 FT-IR (Beaconsfield, UK) spectrophotometer as KBr pellets or thin films. NMR spectra were recorded on a Bruker Avance 500 Spectrometer (Faelanden, Switzerland ) ^1^H at 500 MHz and ^13^C at 125 MHz. Deuterated chloroform (CDCl_3_) and deuterated methanol (MeOD) were used as solvents (Merck, Darmstadt, Germany). Electrospray ionization mass spectra (ESIMS) were obtained using a JEOL JMS-HX-110 (Peabody, MA, USA) mass spectrometer. Silica gel, 70–230 mesh, was used for column chromatography, and thin-layer chromatography (TLC) was performed on Merck pre-coated silica gel 60 F_254+365_. Acetylthiocholine iodide (ATChI), acetylcholinesterase (AChE) from electric eels (type V-S), 5,5V-dithiobis[2-nitrobenzoic acid] (DTNB), bovine serum albumin fraction V (0.1%), physostigmine were obtained from Sigma-Aldrich (St. Louis, MO, USA). Tris-hydrochloric acid buffer (50 mM) at pH 8.0 (Sigma-Aldrich) was used to prepare enzyme solutions (R&M Chemicals, Essex, UK). Industrial grade solvents were used for extraction processes, and analytical grade solvents (Merck) were used for chromatography and recrystallization.

### 3.2. Plant Materials

*Tinospora crispa* vines were collected in Bentong, Pahang, identified by the botanist, Berhaman Ahmad (Universiti Malaysia Sabah), and a voucher specimen FRI54832 deposited at the Forest Research Institute Malaysia (KEP).

### 3.3. Extraction and Chromatography

Powdered, dried *Tinospora crispa* vines (5.0 kg) were first de-fatted by percolation with hexane andfiltration (fraction A). The plant material was dried, then extracted with MeOH-H_2_O (4:1) by sonication for three cycles of 30 min each and then filtered to give a brown extract (fraction B) which was concentrated under low pressure. The concentrated extract was acidified with H_2_SO_4_ to pH 2 and then partitioned with CHCl_3_. The CHCl_3_ phase was collected and concentrated, leaving a brownish gummy residue (fraction C) of non-alkaloidal components. The aqueous phase was basified with NH_4_OH to pH 10 and extracted with MeOH-CHCl_3_ (3:1) to yield a dark brown gummy residue (fraction D) most likely containing alkaloids. Fraction D (25.0 g) was chromatographed over a column of silica gel slurry packed in CHCl_3_. Elution was initiated with CHCl_3_ and progressed through the solvent series of 10%, 15%, 30%, 50%, and 70% MeOH in CHCl_3_, 100% MeOH, and 10% to 15% of NH_4_OH in MeOH to afford nine subfractions (F1–F9). Subfraction 5 was subjected to silica gel column chromatography eluting with MeOH-CHCl_3_ (6:4) to yield compounds **1** (4.5 mg), **2** (5.5 mg) and **3** (10 mg). Subfraction 8 was eluted with MeOH-CHCl_3_ gradually. After reaching 50%, a few drops of NH_4_OH were added to yield compound **4** (8 mg). Subfraction 2 was eluted with EtOAc-Hex (7:3) to yield compounds **5** (3.5 mg) and **6** (45 mg). Subfraction 3 was eluted with CHCl_3_-MeOH (3:1) to yield compound **7** (40 mg).

### 3.4. Spectroscopic Data

Compound **1**: R*_f_* 0.40 (CHCl_3_-MeOH-Hex 7:3:1). IR (KBr): 3449, 1584, 1637, 1091 cm^−1^. UV/Vis λ_max_ (MeOH) nm (log *ε*): 213, 291. ^1^H-NMR (500 MHz, MeOD): 3.81 (3H, s, 9-OCH_3_), 4.07 (3H, s, 2-OCH_3_), 4.21 (3H, s, 8-OCH_3_), 6.52 (1H, dd, *J =* 8.0, 2.0, H-11), 6.56 (1H, d, *J =* 2.0, H10), 6.86 (1H, d, *J =* 8.5, H-12), 7.28 (1H, br s, H-1), 7.35 (1H, br s, H-3), 7.87 (1H, d, *J =* 6.50, H-5), 7.96 (1H, d, *J =* 6.00, H-6).^ 13^C-NMR (125 MHz, MeOD): 44.2 (8-OCH_3_), 54.9 (9-OCH_3_), 55.0 (2-OCH_3_), 103.8 (C-3), 107.6 (C-1), 111.8 (C-12), 114.7 (C-10), 118.5 (C-11), 21.3 (C-5), 121.5 (C-8a), 127.1 (C-12a), 127.2 (C-4a), 130.31 (C-6), 132.0 (C-14a), 136.9 (C-14), 147.2 (C-9), 147.5 (C-13), 150.4 (C-8), 162.3 (C-2), 163.8 (C-4). HRESIMS: *m/z* (%) 352.0075 [M^+^] (2), 339.0653 (25), 311.0237 (77), 310.0366 (100), 296.0022 (13), 282.0120 (16), 151.0560 (14). Yield (4.5 mg, 0.018%).

Compound **2**: R*_f_* 0.42 (CHCl_3_-MeOH 6:4). IR (KBr): 3371, 1584, 1361, 1090 cm^−1^. UV/Vis λ_max_ (MeOH) nm (log *ε*): 293, 372. ^1^H-NMR (500 MHz, MeOD): 3.23 (1H, t, *J* = 5.25, H-5), 3.95 (3H, s, 2-OCH_3_), 4.07 (3H, s, 9-OCH_3_), 4.85 (1H, t, *J =* 5.90, H-6), 7.05 (1H, s, H-4), 7.49 (1H, s, H-1), 7.65 (1H, d, *J =* 9.10, H-11), 7.77 (1H, d, *J =* 8.00, H-12), 8.44 (1H, s, H-13), 9.39 (1H, s, H-8). ^13^C-NMR (125 MHz, MeOD) 26.4 (C-5), 55.2 (2-OCH_3_), 55.9 (C-6), 60.0 (9-OCH_3_), 110.5 (C-4), 111.2 (C-1), 119.6 (C-13), 119.9 (C-14a), 123.0 (C-8a), 123.2 (C-12), 126.4 (C-4a), 132.2 (C-12a), 133.9 (C-11), 135.4 (C-14), 141.6 (C-8), 142.1 (C-9), 146.6 (C-3), 150.5 (C-2), 151.02 (C-10). ESIMS: *m/z* (%) 324 [M^+^] (10), 311 (8), 226 (16), 175(13), 148 (26), 136 (46), 114 (100). Yield (5.5 mg, 0.022%).

Compound **3**: R*_f_* 0.30 (CHCl3-MeOH 7:3). IR (KBr): 3459, 1639, 1092 cm-1. UV/Vis λ_max_ (MeOH) nm (log ε): 290, 348. ^1^H-NMR (500 MHz, MeOD): 3.29 (2H, t, *J* = 6.25, H5), 3.99 (3H, s, 3-OCH_3_), 4.13 (3H, s, 9-OCH_3_), 4.23 (3H, s, 9-OCH_3_), 4.85 (2H, t, J = 6.45, H-6), 7.05 (1H, s, H-4), 7.59 (1H, s, H-1), 8.03 (1H, d, *J* = 9.10, H-12), 8.14 (1H, d, *J* = 9.15, H-11), 8.67 (1H, s, H-13), 9.77 (1H, s, H-8). ^13^C-NMR (125 MHz, MeOD): 26.4 (C5), 55.3 (3-OCH_3_), 56.3 (C-6), 56.4 (10-OCH_3_), 61.2 (9-OCH_3_), 110.6 (C-4), 111.7 (C-1), 119.2 (C-14a), 119.7 (C-13), 121.8 (C-8a), 123.1 (C-12), 126.6 (C-11), 127.4 (C-4a), 133.8 (C-12a), 138.5 (C-14), 144.2 (C-10), 144.8 (C-8), 146.6 (C-2), 150.5 (C-3), 151.1 (C-3). ESIMS: *m/z* (%) 338 [M+] (10), 279 (75), 165(80), 149 (100), 57(95). Yield (10 mg, 0.04%).

Compound **4**: Mp: 250–251 °C (lit [23] 248 °C). R*_f_* 0.30 (MeOH-NH4OH 9:1).UV/Vis λ_max_ (MeOH) nm (log ε): 221, 274, 310. ^1^H-NMR (500 MHz, MeOD): 2.75 (1H, t, *J* = 12.60, H-7b), 2.88 (1H, dd, *J* = 17.50, 4.05, H-4b), 2.98 (3H, s, N-CH3α), 3.16 (1H, m, H-7b), 3.28 (1H, m, H-4a), 3.36 (3H, s, N-CH3β), 3.59 (1H, m, H-5a), 3.68 (1H, m, H-5b), 3.83 (3H, s, 10-OCH3), 3.84 (3H, s, 2-OCH_3_), 4.23 (1H, d, *J* = 12.30, H-6a), 6.62 (1H, s, H-9), 6.64 (1H, s, H-3), 6.75 (1H, d, *J* = 7.95, H-8). ^13^C-NMR (125 MHz, MeOD): 23.4 (C-4), 30.7 (C-7a), 42.0 (N-CH3α), 52.5 (NCH3β), 54.6 (10-OCH_3_), 55.0 (2-OCH_3_), 61.2 (C-5), 70.4 (C-6a), 108.0 (C-8), 109.3 (C-3), 115.6 (C-9), 119.6 (C-3a), 120.1 (C-11c), 123.2 (C-11b), 124.5 (C-11a), 126.2 (C-8a), 149.3 (C-1), 149.1 (C-11), 150.5 (C-10), 151.4 (C-2). ESIMS: *m/z* (%) 342 [M+] (2), 312 (14), 298 (58), 284 (84), 270 (100), 256 (79), 240 (37). Yield (8.0 mg, 0.32%).

Compound **5**: Mp: 216–217 °C (lit. [15] 218–219 °C). R*_f_* 0.78 (Hex-EA 6:4). IR (KBr): 1658, 2925, 2854 cm^−1^. ^1^H-NMR (500 MHz, CDCl_3_) (*Z* isomer): 2.87 (2H, m, H-4α, H-7α), 2.97 (1H, m, H-4β), 3.27 (1H, m, H-7β), 3.41 (1H, dd, *J* = 12.45, 2.80, H-5α), 3.87 (1H, ddd, *J* = 12.80, 4.60, 1.80, H-5β), 5.09 (1H, dd, *J* = 14.00, 4.40, H-6a), 6.02 (1H, d, *J* = 0.5, OCH_2_O), 6.14 (1H, d, *J* = 1.0, OCH_2_O), 6.61 (1H, s, H-3), 7.2–7.37 (3H, overlapped, H-8, H-9, H10), 8.13 (1H, d, *J* = 9.0, H-11), 8.29 (1H, s, CHO). ^13^C-NMR (125 MHz, CDCl_3_): 31.0 (C-4), 33.6 (C-7), 42.1 (C-5), 49.4 (C-6a), 101.0 (OCH_2_O), 107.5 (C-3), 117.4 (C-11b), 124.7 (C-11c), 126.6 (C-3a), 127.1-128.0 (C-8, C-9, C-10), 128.8 (C-11), 130.6 (C11a), 135.1 (C-7a), 143.3 (C1), 147.1 (C-2), 162.1 (CHO). ^1^H-NMR (500 MHz, CDCl_3_) (*E* isomer): 2.87 (3H, m, H-4α, H-4β, H-7α), 3.15 (2H, m, H-5β, H-7β), 4.51 (1H, ddd, *J* = 12.70, 4.45, 1.80, H-5β), 4.66 (1H, dd, *J* = 14.50, 4.40, H-6a), 6.02 (1H, d, *J* = 0.5 OCH_2_O), 6.15 (1H, d, *J* = 1.0, OCH_2_O), 6.61 (1H, s, H-3), 7.27–7.37 (3H, m-overlapped, H-8, H-9, H10), 8.15 (1H, d, *J* = 9.0, H-11), 8.42 (1H, s, CHO). ^13^C-NMR (125 MHz, CDCl_3_): 29.7 (C-4), 37.7 (C-7), 36.1 (C-5), 53.3 (C-6a), 101.1 (OCH2O),107.5 (C-3), 117.0 (C-11b), 124.1 (C-11c), 126.6 (C-3a), 127.1–128.0 (C-8, C-9, C-10), 128.4 (C-11), 130.4 (C11a), 134.4 (C-7a), 143.1 (C1), 147.4 (C-2), 162.0 (CHO). ESIMS: *m/z* (%) 293[M+] (52), 251(43), 236(27), 235 (100). Yield (3.5 mg, 0.014%).

Compound **6**: Mp: 220–221 °C (lit. [15] 222–224 °C). R*_f_* 0.61 (Hex-EA 7:3). IR (KBr): 1663, 1032 cm^‑1^. ^1^H-NMR (500 MHz, CDCl_3_) (*Z* isomer): 2.75 (2H, m, H-4α,H-7β), 2.91 (1H, m, H-4β), 3.15 (1H, m, H-7α), 3.41 (1H, dd, *J* = 12.50, 2.75, H-5α), 3.67 (3H, s, 1-OCH_3_), 3.82 (1H, ddd, *J* = 12.75, 4.60, 1.80, H-5β), 3.89 (3H, s, 2-OCH_3_), 4.92 (1H, dd, *J* = 14.35, 4.10, H-6a), 6.66 (1H, s, H-3), 7.26–7.34 (3H, overlapped, H-8, H-9, H10), 8.44 (1H, d, *J* = 9.0, H-11), 8.26 (1H, s, CHO). ^13^C-NMR (500 MHz, CDCl_3_): 30.9 (C-4),34.1 (C-7), 42.0 (C-5), 49.4 (C-6a), 56.0 (1-OCH_3_), 60.0 (2-OCH_3_), 111.4 (C-3), 125.2 (C-3a), 127.4 (C-11b), 127.5-127.9 (C-8,C-9,C-10), 128.4 (C-11), 128.6 (C-11c), 131.4 (C11a), 136.1 (C-7a), 145.9 (C1), 152.4 (C-2), 162.1 (CHO). ^1^H-NMR (500 MHz, CDCl_3_) (*E* isomer): 2.75 (3H, m, H-4α, H-4β, H-7β), 3.15 (2H, m, H-7α, H-5α), 3.67 (3H, s, 1-OCH_3_), 3.89 (3H, s, 2-OCH_3_), 4.43 (1H, ddd, *J* = 12.70, 4.50, 3.65, H-5β), 4.51 (1H, dd, *J* = 14.20, 4.00, H-6a), 6.69 (1H, s, H-3), 7.26-7.34 (3H, overlapped, H-8, H-9, H10), 8.41 (1H, d, *J* = 9.0, H-11), 8.26 (1H,s, CHO). ^13^C-NMR (125 MHz, CDCl_3_): 29.6 (C-4), 36.1 (C-5), 37.9 (C-7), 53.4 (C-6a), 56.0 (1-OCH_3_), 60.0 (2-OCH_3_), 111.7 (C-3), 124.7 (C-3a), 127.4 (C-11b), 127.5–127.9 (C-8,C-9,C-10), 128.6 (C-11), 129.5 (C-11c), 131.1 (C11a), 135.4 (C-7a), 145.7 (C1), 152.6 (C-2), 161.9 (CHO). ESIMS: *m/z* (%) 309 [M+] (90), 250(100), 237(11). Yield (45.0 mg, 0.18%).

Compound **7**: Mp 89–90 °C (lit. [16] 91 °C). R*_f_* 0.60 (Hex-EA 7:3). IR (KBr): 3228, 1651, 1031 cm^−1^. ^1^H-NMR (500 MHz, MeOD): 2.77 (1H, t, *J* = 7.6, H-2), 3.49 (1H, t, *J* = 7.20, H-1), 3.92 (3H,s, 6'-OCH_3_), 6.42 (1H, d, *J* = 15.70, H-2'), 6.73 (2H, d, *J* = 8.60, H5, H-7), 6.81 (1H, d, *J* = 8.2, H-8'), 7.04 (1H, dd, *J* = 8.20, 1.90, H-9'), 7.06 (2H, d, *J* = 8.5, H-4, H-8), 7.13 (1H, d, *J* = 1.90, H-5'), 7.45 (1H, d, *J* = 15.7, H-3'). ^13^C-NMR (125 MHz, MeOD): 32.8 (C-2), 39.6 (C-1), 53.4 (6'-OCH_3_), 106.6 (C-5'), 113.3 (C-5, C-7), 113.5 (C-8'), 115.8 (C-2'), 120.3 (C-8), 125.3 (C-4'), 127.8 (C-4, C-8), 128.4 (C-3), 139.1 (C-3'), 146.3 (C-7'), 146.9 (C-6'), 154.0 (C-6), 166.2 (C-1'). ESIMS: *m/z* (%) 313[M+] (21), 193 (26), 192(41), 177(100), 145 (12), 120 (15). Yield (40 mg, 0.16%).

### 3.5. Acetylcholinesterase (AChE) Inhibitory Assay

The three compounds were tested for AChE inhibiting activity using a modified Ellman’s colorimetric method [24]. Three buffers were prepared for the assay: buffer A (50 mM Tris-HCl, pH 8), buffer B (50 mM, pH 8, containing 0.1% bovine serum albumin), and buffer C (50 mM Tris-HCl, pH 8, containing 0.1 M NaCl and 0.02 M MgCl_2_∙6H_2_O). In the 96-well plates, 25 μL of 15 mM acetylthiocholine iodide (ATChI) in water, 125 μL of 3 mM DTNB in buffer C, 50 μL of buffer B, and 25 μL of isolated compounds dissolved in MeOH at concentrations ranging from 62.5 to 1000 μg/mL were added. Then 25 μL of 0.22 UmL^−1^ of AChE was added and the absorbance measured at 405 nm was read every 10 min twice. To avoid false positive results, a parallel test omitting the DTNB was done. and none of the test substances were found to interact with thiocholine formed during the assay. Physostigmine served as the positive control. All assays were performed in duplicate in a 96-well microplate reader (Tecan Infinite 200 Pro). The percentage inhibition was calculated using the formula (1):

% Inhibition = [(E − S)/S] × 100
(1)

where E is the enzyme activity without the test compound, and S is the enzyme activity with the test compound. IC_50_ values were calculated by plotting percentage inhibition against the extract concentration (Table 2).

## 4. Conclusions

The study on AChE inhibitory activity of isolated compounds showed that it was influenced by the alkaloid skeleton but especially by the ring substituents. Three different types of alkaloids were isolated, namely, aporphine, oxoaporphine and quaternary protoberberines. The AChE inhibitory results showed that the most active compound was the least polar one, columbamine. One of the outcomes of this investigation is that it suggests that the alkaloids are suitable chemical markers for *T. crispa*, as well as the genus *Tinospora*, due to their strong presence within this genus. The results of the present investigation further point to the potential of this genus as a source of new AChE inhibitors.
